# Coniferaldehyde Reverses PGK1–3-Phosphoglycerate–L-Serine Metabolic Reprogramming in Hindlimb Ischemia

**DOI:** 10.3390/metabo16100756

**Published:** 2026-10-09

**Authors:** Yun Dong, Chunbo Ma, Xueli Wang, Jiaping Han, Zina Zhu, Suwen Hu, Zixuan Hu, Liangrong Wang

**Affiliations:** 1 Department of Anesthesiology, Ninghai First Hospital, Ningbo 315600, China; 2 Department of Anesthesiology, The First Affiliated Hospital of Wenzhou Medical University, Wenzhou 325000, Chinazin9531@163.com (Z.Z.);; 3 The First Clinical Medical College, Wenzhou Medical University, Wenzhou 325000, China; 4 Department of Lung Cancer Surgery, Tianjin Medical University General Hospital, Tianjin 300052, China

**Keywords:** peripheral artery disease, hindlimb ischemia, metabolic reprogramming, endothelial metabolism, phosphoglycerate kinase 1, 3-phosphoglycerate, serine biosynthesis, coniferaldehyde, angiogenesis

## Abstract

**Highlights:**

**What are the main findings?**

- In ischemic gastrocnemius, coniferaldehyde reversed the HLI-associated increase in *Pgk1* expression and reduced 3-phosphoglycerate and serine.
- AlphaFold3 modeling, molecular dynamics simulation, and CETSA supported a potential coniferaldehyde–PGK1 interaction.
- Conditioned medium from coniferaldehyde-treated hypoxic skeletal muscle cells improved several endothelial readouts, whereas direct 3-phosphoglycerate supplementation reversed these effects.

**What are the implications of the main findings?**

- Coniferaldehyde-associated metabolic remodeling in skeletal muscle may contribute to endothelial protection and vascular repair.
- These findings support further investigation of the therapeutic potential of coniferaldehyde for ischemic vascular disease.

**Abstract:**

**Background/Objectives:** Ischemic skeletal muscle undergoes metabolic remodeling that may influence vascular repair. We investigated whether coniferaldehyde (CA) regulates a phosphoglycerate kinase 1 (PGK1)–3-phosphoglycerate (3PG)–L-serine axis in mouse hindlimb ischemia (HLI). **Methods:** Gastrocnemius tissue from Sham, HLI, and HLI+CA mice (*n* = 6/group) underwent bulk RNA sequencing and untargeted metabolomics. Public single-cell data and hypoxic human skeletal muscle cells (HSkMCs) were used to examine PGK1. AlphaFold3 modeling, molecular dynamics simulation, and a cellular thermal shift assay (CETSA) were used to evaluate a potential CA–PGK1 interaction. HSkMC-conditioned media were applied to human umbilical vein endothelial cells (HUVECs), with 3PG supplementation where indicated. **Results:** Amino-acid biosynthesis and glycine/serine/threonine metabolism were enriched in HLI muscle. 3PG increased in HLI versus Sham (*p* = 0.0170) and decreased in HLI+CA versus HLI (*p* = 0.0358); L-serine followed the same pattern (*p* = 0.0274 and *p* = 0.0428, respectively). *Pgk1* expression also increased in HLI versus Sham (*p* = 0.00348) and decreased in HLI+CA versus HLI (*p* = 0.00249). Hypoxia increased HSkMC PGK1 together with extracellular 3PG and serine, whereas CA attenuated all three. Modeling predicted a stable CA–PGK1 complex, and CETSA supported a potential CA–PGK1 interaction in cell lysates. Conditioned medium from CA-treated hypoxic HSkMCs improved HUVEC viability, redox, tube-formation, and wound-closure readouts, reduced reactive oxygen species, and increased glutathione peroxidase 4 (GPX4) and solute carrier, family 7 member 11 (SLC7A11); 3PG supplementation reversed these effects. **Conclusions:** CA modulates a PGK1-linked 3PG/L-serine metabolic axis, while 3PG exposure altered endothelial phenotypes in the conditioned-medium model.

## 1. Introduction

Lower-extremity peripheral artery disease (PAD) is a heterogeneous atherosclerotic disorder that ranges from asymptomatic disease to chronic symptomatic PAD, chronic limb-threatening ischemia, and acute limb ischemia [1]. In 2019, an estimated 113 million adults aged 40 years or older were living with PAD worldwide, with most attributable disability linked to modifiable risk factors [2]. Despite contemporary risk-factor management and limb-directed therapy, PAD remains underrecognized and continues to cause mobility loss, tissue loss, amputation, and excess cardiovascular mortality [1,2,3].

The biological burden of PAD is not explained by conduit-vessel stenosis alone. Chronically underperfused skeletal muscle develops mitochondrial, oxidative, structural, transcriptomic, and proteomic abnormalities that contribute to exercise limitation [4,5]. Experimental HLI is widely used to interrogate ischemic injury and perfusion recovery, although it does not reproduce the chronic atherosclerotic biology of human PAD in full. In both experimental ischemia and human PAD, glycolytic capacity and metabolic flexibility have emerged as potentially modifiable components of muscle pathology; for example, enhancing PFKFB3-dependent glycolysis improved muscle function and blood flow in an ischemic limb model [6]. Conversely, conventional growth-factor- and cell-based therapeutic angiogenesis has not consistently translated into major clinical benefit [7]. These observations motivate approaches that address the metabolic state of the ischemic tissue niche rather than treating vessel growth as an isolated endothelial program.

Recovery from limb ischemia depends on communication among skeletal-muscle, stromal, immune, and vascular cells [8,9,10]. Endothelial cells are metabolically active: glycolysis, fatty-acid metabolism, amino-acid metabolism, and mitochondrial pathways instruct migration, proliferation, redox homeostasis, and vessel maturation [11,12,13]. PFKFB3-driven glycolysis is causally required for endothelial sprouting, while partial glycolytic inhibition can restrain pathological angiogenesis [14,15], illustrating that the consequences of metabolic intervention are context-dependent. Single-cell studies further demonstrate substantial endothelial metabolic plasticity [16]. Of particular relevance, de novo serine synthesis through phosphoglycerate dehydrogenase (PHGDH) supports endothelial heme synthesis, mitochondrial respiration, proliferation, and angiogenesis [17]. Skeletal-muscle metabolic programs can also influence angiogenesis and revascularization after HLI [18]. Recent single-cell and spatial analyses of human PAD muscle identified altered endothelial diversity, macrophage activation, and remodeled cell–cell communication [19], while cell-specific metabolic reprogramming in ischemic muscle can reshape the inflammatory secretory milieu and endothelial revascularization [20]. Thus, cell-type-specific metabolism and metabolic crosstalk provide a plausible framework for understanding vascular repair in ischemic muscle.

Hypoxia-inducible factors coordinate adaptation to limited oxygen by shifting glucose utilization toward glycolysis and inducing multiple glycolytic genes [21,22,23]. Phosphoglycerate kinase 1 (PGK1), a canonical hypoxia-responsive enzyme, catalyzes the reversible conversion of 1,3-bisphosphoglycerate and ADP to 3-phosphoglycerate (3PG) and ATP [24]. The resulting 3PG is a branch-point metabolite: PHGDH oxidizes 3PG to 3-phosphohydroxypyruvate, phosphoserine aminotransferase 1 (PSAT1) generates 3-phosphoserine, and phosphoserine phosphatase (PSPH) produces L-serine [25,26,27,28,29]. Serine metabolism contributes to mitochondrial redox control during hypoxia [30], and PGK1 also has noncanonical functions in other disease contexts [31]. Whether this pathway participates in ischemic skeletal-muscle remodeling, and whether 3PG and serine influence vascular repair through distinct endothelial and inflammatory routes, remains unknown.

Coniferaldehyde (CA; 4-hydroxy-3-methoxycinnamaldehyde) is a naturally occurring phenolic aldehyde with reported antioxidant and anti-inflammatory activity. In Alzheimer’s disease, macrophage, and neuroinflammation models, CA activated nuclear factor erythroid 2-related factor 2 (Nrf2)-linked defenses and attenuated oxidative or inflammatory injury [32,33,34,35]. In the mouse HLI model, CA improved hindlimb perfusion and reduced ischemia scores (Figure S1). The metabolic effects of CA in ischemic skeletal muscle, however, remain incompletely defined.

Here, we combined untargeted metabolomics, bulk RNA sequencing, single-cell RNA sequencing, hypoxic HSkMC experiments, and an HSkMC-conditioned-medium/HUVEC model. We examined CA-associated changes in a candidate ischemia-associated PGK1–3PG–L-serine axis, quantified extracellular 3PG and serine in HSkMC cultures, and used direct 3PG supplementation to examine endothelial phenotypes.

## 2. Materials and Methods

### 2.1. Study Design

The study integrated mouse HLI phenotyping, gastrocnemius metabolomics and transcriptomics, public single-cell transcriptomics, structural modeling and CETSA, HSkMC experiments, and HSkMC-conditioned-medium/HUVEC assays. The formal matched analysis set comprised Sham, HLI, and HLI+CA mice (*n* = 6/group); the same 18 animal identifiers were represented in the phenotyping, transcriptomic, and metabolomic datasets. Figure S1 presents the complete phenotyping cohort (*n* = 8/group).

### 2.2. Animals

Male C57BL/6J mice (8–10 weeks old; GemPharmatech Co., Ltd., Nanjing, China) were acclimated for 3 days and randomly assigned, with body weight balanced, to Sham, HLI, or HLI+CA groups. Mice were housed under specific-pathogen-free conditions on a 12 h light/12 h dark cycle with free access to food and water. Under isoflurane anesthesia, HLI was induced by proximal and distal ligation of the right femoral artery followed by transection between the ligatures; Sham mice underwent the same surgical exposure without arterial ligation or transection. Randomization and blinded assessment were used in the animal experiments.

CA was obtained from MedChemExpress (catalog HY-N2535; batch 241319; Monmouth Junction, NJ, USA). A 20 mg/mL DMSO stock was diluted to a 2 mg/mL dosing formulation containing 10% DMSO, 40% polyethylene glycol 300, 5% Tween 80, and 45% physiological saline. The 2 mg/mL CA formulation was administered intraperitoneally at 10 µL/g body weight, equivalent to 20 mg/kg, once daily from Day −3 through Day 7; Sham and HLI mice received the same volume and schedule of vehicle. At the experimental endpoint, mice were euthanized by cervical dislocation under anesthesia.

Hindlimb perfusion was measured with a Perimed laser Doppler system (Perimed AB, Stockholm, Sweden) on postoperative Days 0, 3, and 7. The perfusion ratio was calculated as the signal from the ischemic limb divided by that from the contralateral nonischemic limb (I/NI), and recovery was expressed relative to the Day 0 value. Limb ischemia was scored daily from Day 0 through Day 7 on a 0–3 scale: 0, normal skin color; 1, pale paw; 2, cyanotic or necrotic toes; and 3, whole-foot necrosis or autoamputation. Body weight was recorded from Day −3 through Day 7. Figure S1 presents eight mice per group; the matched omics analysis included six mice per group. Daily monitoring included body weight and limb condition.

### 2.3. Untargeted Metabolomics

Gastrocnemius samples from Sham, HLI, and HLI+CA mice (*n* = 6/group) were analyzed by Honsunbio Technology Co., Ltd. (Shanghai, China) using positive- and negative-ion liquid chromatography–mass spectrometry (LC–MS). Data were converted to mzXML with ProteoWizard MSConvert version 3.0.8789. Feature detection, retention-time correction, grouping, and alignment were performed separately by ion mode using XCMS version 3.12.0 and the centWave method (bandwidth = 2, mass tolerance = 15 ppm, peak width = 5–30 s, *m*/*z* width = 0.015, and minimum *m*/*z* difference = 0.01). Features with a quality-control relative standard deviation >30% were excluded. A total of 871 annotated features were retained and transformed as log2(intensity + 1). Metabolites were annotated against the Human Metabolome Database (HMDB), MassBank, LIPID MAPS, mzCloud, Kyoto Encyclopedia of Genes and Genomes (KEGG), and an in-house standard library. The analyzed 3PG and L-serine features were assigned Metabolomics Standards Initiative (MSI) level 2 [36].

Differential abundance was analyzed with limma version 3.62.2 using empirical-Bayes moderation [37]. Volcano plots highlighted features with nominal *p* < 0.05 and an absolute fold change ≥1.5, and Benjamini–Hochberg false-discovery rates (BH-FDRs) were calculated across the 871 features separately for each contrast. Partial least-squares discriminant analysis (PLS-DA) was performed with mixOmics version 6.30.0 [38], and pairwise two-component models were refitted under 499 label permutations. Principal component analysis (PCA; Figure S2) and permutational multivariate analysis of variance (PERMANOVA) used feature-wise scaled log-transformed abundances. PERMANOVA evaluated group differences using 999 permutations [39], and PERMDISP assessed multivariate dispersion using 999 permutations. For exploratory KEGG pathway enrichment, features selected by nominal *p* < 0.05 and variable importance in projection >1 were tested using a hypergeometric test with BH correction across pathways [40].

### 2.4. Bulk RNA Sequencing

Gastrocnemius samples from Sham, HLI, and HLI+CA mice (*n* = 6/group) were processed by Honsunbio Technology Co., Ltd. (Shanghai, China) for RNA extraction, library preparation, and sequencing. Total RNA was extracted with TransZol Up (TransGen Biotech Co., Ltd., Beijing, China) by tissue homogenization, chloroform phase separation, isopropanol precipitation, ethanol washing, and dissolution in RNase-free water. Polyadenylated RNA was then enriched with oligo (dT) magnetic beads, and libraries were prepared with the VAHTS Universal V8 RNA-seq Library Prep Kit for Illumina (Vazyme, Nanjing, China). Libraries were sequenced on an Illumina NovaSeq platform (Illumina, San Diego, CA, USA) using paired-end 150 bp reads. Reads were processed with fastp version 0.22.0 and aligned to the Ensembl *Mus musculus* GRCm39 release 106 reference genome with STAR version 2.7.7a. Gene expression was expressed as log2(transcripts per million [TPM] + 1) and analyzed with limma version 3.62.2 using a no-intercept group design, contrasts of HLI versus Sham, HLI+CA versus HLI, and HLI+CA versus Sham, empirical-Bayes moderation, and BH adjustment across genes for each contrast. This model used the 18,159-gene log-transformed expression matrix. Transcriptomic PLS-DA was performed with mixOmics version 6.30.0 (plsda, ncomp = 2) using gene-wise scaled values for 1330 genes from the vendor low-expression-filtered TPM matrix. PCA of the same gene-wise scaled 1330-gene TPM matrix is shown in Figure S3. Supplementary Data S3 links the 18 RNA sample identifiers in Supplementary Data S1 to their groups and matched metabolomic samples.

### 2.5. Single-Cell RNA-Sequencing Analysis

Public gastrocnemius single-cell RNA-sequencing data from GSE215922 were reanalyzed [8]. Cells from ten mice per condition were pooled into one 10× Genomics (Pleasanton, CA, USA) library for Sham and one for HLI. After quality control, 15,958 cells were analyzed with Seurat [41] using the source-study cell annotations. Uniform manifold approximation and projection (UMAP) was used to display cell identities and *Pgk1* expression, and mean expression and the percentage of *Pgk1*-positive cells were summarized by cell type and condition.

### 2.6. Cell Culture

Human skeletal muscle cells (HSkMCs) and human umbilical vein endothelial cells (HUVECs) were obtained from BDBIO (Hangzhou, China) and confirmed to be free of mycoplasma. HSkMCs were maintained in Skeletal Muscle Cell Growth Medium (Merck, Darmstadt, Germany), formulated for human skeletal muscle cell expansion. HUVECs were maintained in Dulbecco’s modified Eagle medium supplemented with 10% fetal bovine serum; DMEM, fetal bovine serum, phosphate-buffered saline, and trypsin–EDTA were obtained from Gibco (Waltham, MA, USA). Cells were cultured at 37 °C in a humidified atmosphere containing 5% CO_2_.

Normoxic cultures were maintained at atmospheric oxygen, whereas hypoxia was established at 1% O2. HSkMCs were cultured for 48 h under normoxia, hypoxia, or hypoxia with 5 µM CA.

### 2.7. HSkMC–HUVEC Indirect Co-Culture Assay

For conditioned-medium experiments, HSkMCs were washed with phosphate-buffered saline after the initial 48 h treatment and cultured for a further 48 h before conditioned medium (CM) was collected. CM was centrifuged at 12,000 rpm for 5 min, mixed 1:1 with complete HUVEC medium, and applied to HUVECs. Where indicated, 3PG was prepared at 200 µM in HUVEC medium before mixing, yielding 100 µM in the final recipient mixture. Five conditions were compared: normoxic-donor CM, normoxic-donor CM plus 3PG, hypoxic-donor CM, CA-treated hypoxic-donor CM, and CA-treated hypoxic-donor CM plus 3PG.

HUVEC metabolic activity after 48 h of CM treatment was assessed using Cell Counting Kit-8 (APExBIO, Houston, TX, USA). Reduced glutathione (GSH) and malondialdehyde (MDA) were measured using commercial kits (Beyotime, Shanghai, China) according to the manufacturer’s instructions. For tube formation, HUVECs were seeded on Matrigel (Selleck, Houston, TX, USA) and incubated for 6 h; total tube length was quantified with ImageJ (v1.54r; Wayne Rasband, National Institutes of Health, Bethesda, MD, USA). For the scratch assay, a linear wound was created with a sterile pipette tip; images were acquired at 0, 24, 48, and 72 h, and wound closure was quantified at 72 h. Each experiment was performed with three independent biological replicates. For tube-formation and scratch assays, three randomly selected fields per biological replicate were averaged before statistical analysis.

Intracellular reactive oxygen species (ROS) in HUVECs were assessed using the Reactive Oxygen Species Assay Kit with Diluent (Beyotime). After treatment, cells were loaded with 2′,7′-dichlorodihydrofluorescein diacetate (DCFH-DA) according to the manufacturer’s protocol, washed to remove extracellular probe, and analyzed using the FITC channel on a NovoCyte 2000R flow cytometer (Agilent Technologies, Inc., Santa Clara, CA, USA). Data were processed using NovoExpress software (version 1.5.0).

### 2.8. Quantitative Reverse-Transcription PCR

Total RNA was reverse-transcribed using a commercial reverse-transcription kit (Vazyme, Nanjing, China), and quantitative PCR was performed with SYBR Green reagents (Vazyme, Nanjing, China) according to the manufacturer’s instructions. Relative *PGK1* mRNA expression was normalized to *GAPDH* and calculated using the 2^−ΔΔCt^ method.

### 2.9. Western Blotting

Cellular proteins were separated by sodium dodecyl sulfate–polyacrylamide gel electrophoresis (SDS–PAGE) and transferred to polyvinylidene difluoride membranes (Millipore, Billerica, MA, USA). Membranes were blocked and incubated with primary antibodies overnight at 4 °C, followed by horseradish peroxidase-conjugated secondary antibodies for 1 h at room temperature. Primary and secondary antibodies were obtained from Proteintech (Wuhan, China). Bands were visualized using enhanced chemiluminescence (Thermo Fisher Scientific, Waltham, MA, USA), and PGK1, glutathione peroxidase 4 (GPX4), and solute carrier family 7 member 11 (SLC7A11) abundance was normalized to β-tubulin. Densitometry was performed using ImageJ version 1.52.

### 2.10. 3-Phosphoglycerate and Serine Assays

3PG and serine concentrations in blank medium and HSkMC culture supernatants were measured using commercial ELISA kits (EK-Bioscience and QIYBO, Shanghai, China, respectively), according to the manufacturers’ instructions. Concentrations were calculated from the corresponding standard curves and expressed in µM.

### 2.11. Structural Modeling and Molecular Dynamics Simulation

The PGK1–CA complex was predicted with AlphaFold3 using 10 random seeds, which generated 50 candidate conformations. The highest-scoring conformation was selected and visualized with PyMOL version 3.1.5.1 (Schrödinger, New York, NY, USA). A 100 ns molecular dynamics simulation was used to evaluate root-mean-square deviation (RMSD), root-mean-square fluctuation (RMSF), radius of gyration, solvent-accessible surface area (SASA), protein–ligand hydrogen bonds, and the free-energy landscape.

### 2.12. Cellular Thermal Shift Assay (CETSA)

For the cellular thermal shift assay, HSkMC lysates were incubated with vehicle or CA and exposed to a temperature gradient from 37 to 64 °C. After centrifugation, soluble PGK1 in the supernatant was detected by Western blotting.

### 2.13. Statistical Analysis

Data were analyzed using R version 4.4.2 (R Foundation for Statistical Computing, Vienna, Austria) and GraphPad Prism version 9.0 (GraphPad Software, Boston, MA, USA) and are presented as mean ± standard deviation (SD). Comparisons between two groups were performed using two-tailed unpaired Student’s *t*-tests, and multiple-group comparisons were performed using one-way analysis of variance (ANOVA) followed by Dunnett’s multiple-comparisons test. For prespecified gene–metabolite pairs, Pearson and Spearman correlation coefficients and ordinary least-squares regressions were calculated across the matched samples. For non-omics assays, *p* < 0.05 was the stated significance threshold. Omics *p* values were distinguished from BH-FDR values.

## 3. Results

### 3.1. Coniferaldehyde Reverses Selected HLI-Associated Metabolite Changes

**Figure 1:**
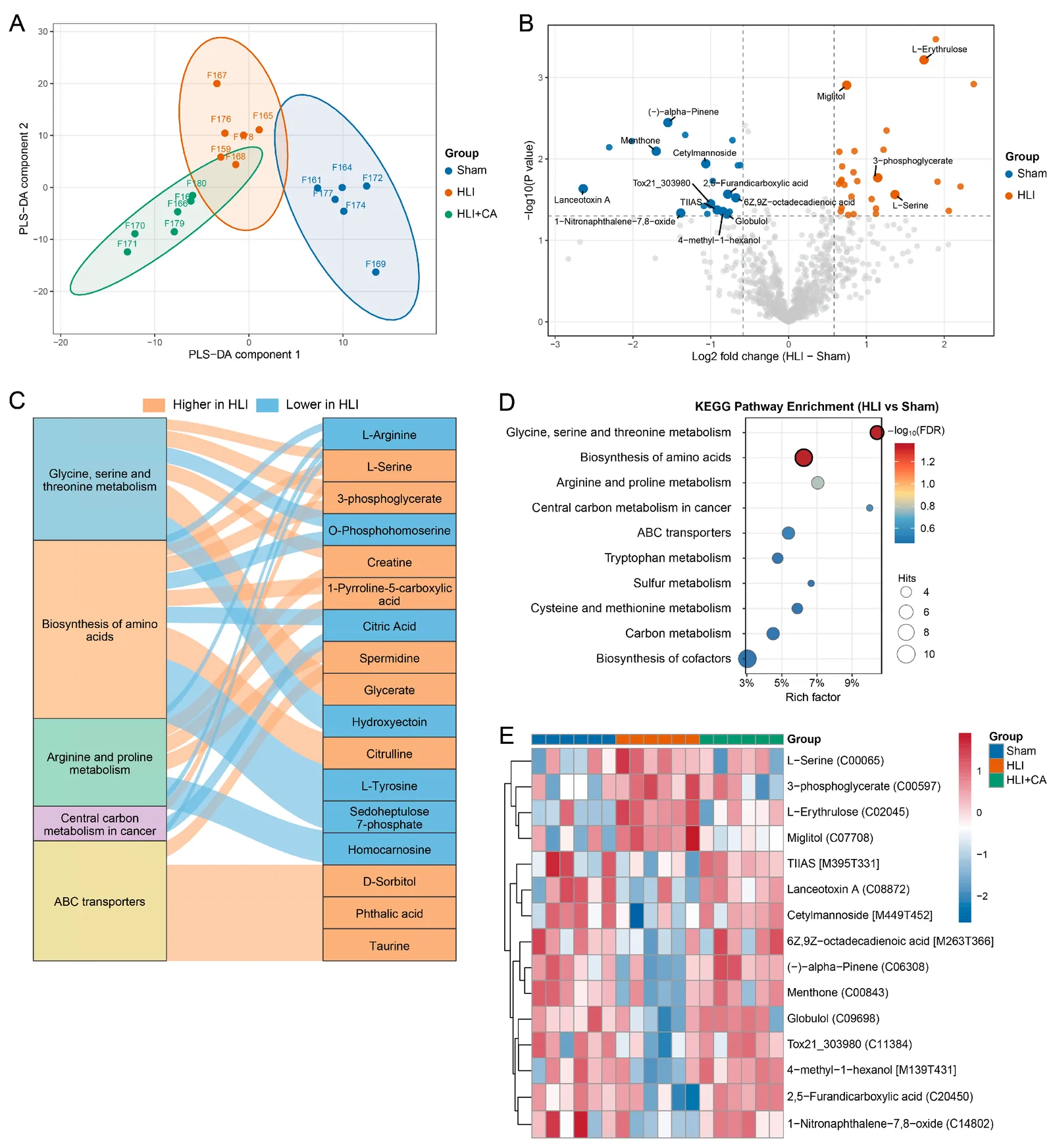
Coniferaldehyde reverses selected HLI-associated metabolite changes in mouse gastrocnemius. (**A**) Partial least-squares discriminant analysis (PLS-DA) scores for Sham, hindlimb ischemia (HLI), and HLI treated with coniferaldehyde (HLI+CA); *n* = 6 mice/group. Ellipses show within-group dispersion. Global permutational multivariate analysis of variance (PERMANOVA): R^2^ = 0.1419, *p* = 0.064; all pairwise BH-adjusted *p* values ≥ 0.177. (**B**) Volcano plot for HLI versus Sham. Orange and blue features meet nominal *p* < 0.05 and |fold change| ≥ 1.5 and are higher or lower in HLI, respectively. Gray features do not meet these thresholds. (**C**) Sankey plot linking enriched Kyoto Encyclopedia of Genes and Genomes (KEGG) pathways to representative metabolites; orange and blue flows denote metabolites higher and lower in HLI versus Sham, respectively. (**D**) KEGG pathway-enrichment bubble plot. Point size denotes the number of hits, position denotes the rich factor, and color denotes −log10(BH-FDR). (**E**) Row-z-scored heatmap of selected metabolites.

We first profiled gastrocnemius metabolites to examine the tissue response to HLI and CA treatment. Exploratory PLS-DA showed clustering of the Sham, HLI, and HLI+CA samples along the first two components (Figure 1A). Global PERMANOVA yielded R^2^ = 0.1419 and *p* = 0.064. Unsupervised PCA showed overlapping group distributions (Figure S2).

The volcano plot highlighted nominally differential metabolite features between HLI and Sham (Figure 1B). Relative to Sham, 3PG and L-serine increased in HLI (nominal *p* = 0.0170 and *p* = 0.0274; BH-FDR = 0.6126 and 0.6845, respectively). In HLI+CA versus HLI, 3PG and L-serine decreased (nominal *p* = 0.0358 and *p* = 0.0428; BH-FDR = 0.6426 and 0.6777, respectively); the corresponding abundance ratios were 0.50 (95% confidence interval [CI], 0.27–0.95) and 0.42 (95% CI, 0.18–0.97). These metabolite comparisons did not meet BH-FDR < 0.05, and the 95% CIs were pointwise rather than multiplicity-adjusted. The Sankey plot connected the altered metabolites with their enriched KEGG pathways (Figure 1C). Biosynthesis of amino acids and glycine, serine, and threonine metabolism were the top enriched pathways in HLI versus Sham (both *p* = 9.00 × 10^−4^; Figure 1D and Table 1). A z-score heatmap showed coordinated HLI-associated increases in 3PG, L-serine, L-erythrulose, and miglitol, and decreases after CA treatment (Figure 1E).

### 3.2. Bulk Transcriptome and Metabolome Integration Examines a Candidate PGK1–3PG–L-Serine Axis in HLI

**Figure 2:**
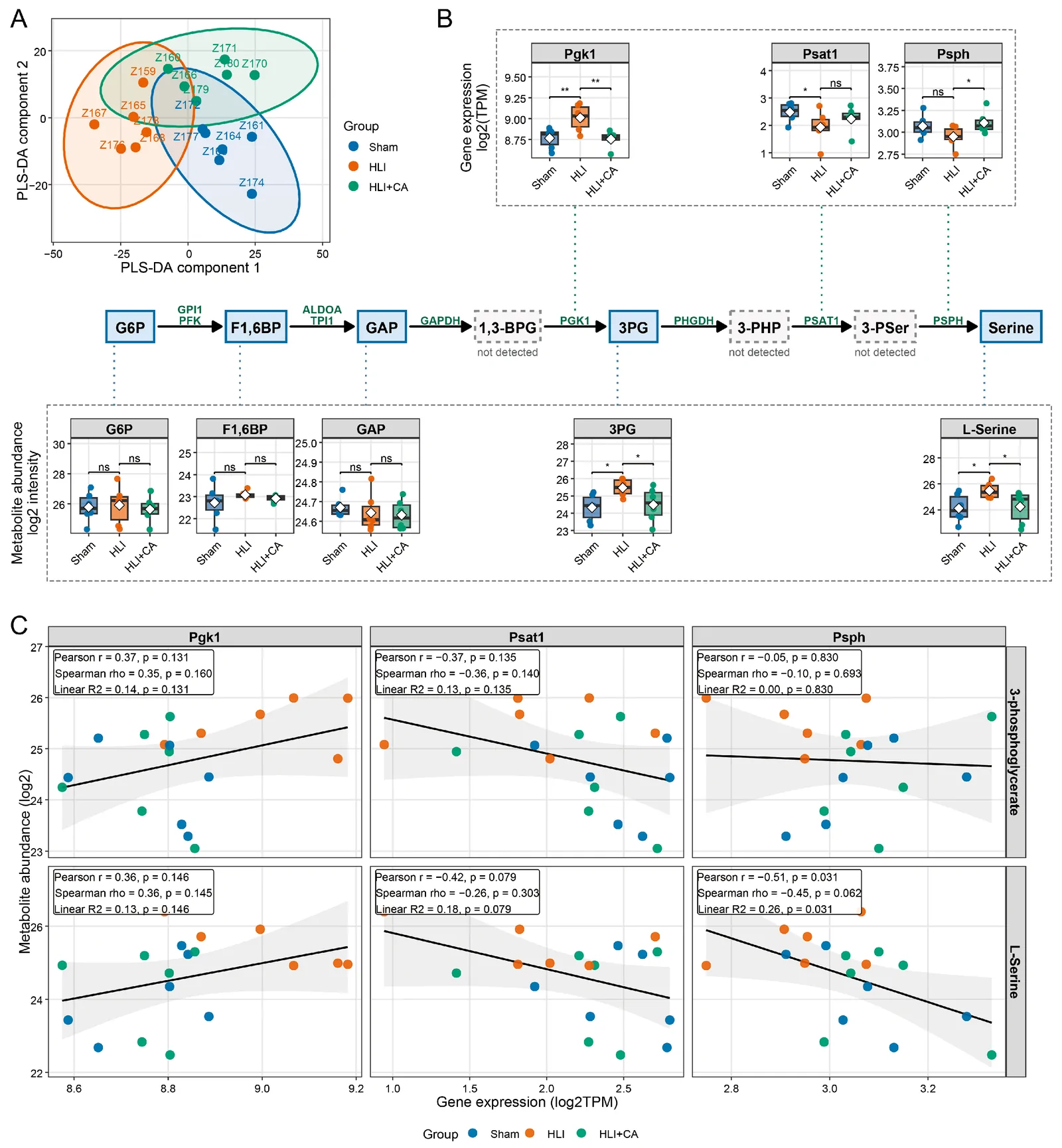
Integrated transcriptomic and metabolomic analysis examines a candidate PGK1–3PG–L-serine axis. (**A**) Transcriptomic PLS-DA scores for Sham, HLI, and HLI+CA (*n* = 6 mice/group). Global PERMANOVA: R^2^ = 0.2600, *p* = 0.002; pairwise BH-adjusted *p* = 0.009 for Sham versus HLI, 0.0165 for HLI versus HLI+CA, and 0.161 for Sham versus HLI+CA. (**B**) Glycolytic branch from 1,3-bisphosphoglycerate (1,3-BPG) through 3-phosphoglycerate (3PG) to L-serine, with gene expression shown as log2(TPM+1) and metabolite abundance as log2(intensity+1). Boxes show the interquartile range, center lines the median, diamonds the mean, whiskers the recorded range, and points individual mice. The nodes for 1,3-BPG, 3-phosphohydroxypyruvate (3-PHP), and 3-phosphoserine (3-PSer) were not detected in the untargeted LC–MS dataset. (**C**) Exploratory pooled correlations across 18 matched mouse samples, without group adjustment. Lines show ordinary least-squares fits and shaded regions 95% confidence intervals. Pearson r, Spearman ρ, R^2^, and *p* values are displayed in each panel. Gene/metabolite annotations use nominal *p* values: * *p* < 0.05; ** *p* < 0.01; ns, nominal *p* ≥ 0.05. Nominal significance does not imply BH-FDR significance.

Exploratory transcriptomic PLS-DA showed groupwise clustering of the Sham, HLI, and HLI+CA samples (global PERMANOVA: R^2^ = 0.2600, *p* = 0.002; Figure 2A). The corresponding unsupervised PCA is provided in Figure S3.

We next examined the glycolytic branch from 1,3-bisphosphoglycerate through 3PG to serine (Figure 2B). *Pgk1* was higher in HLI than Sham (*p* = 0.00348) and lower in HLI+CA than HLI (*p* = 0.00249; expression ratio, 0.84; 95% CI, 0.75–0.93). *Psat1* was lower in HLI than Sham (*p* = 0.0484), and *Psph* was higher in HLI+CA than HLI (*p* = 0.0378). Glucose 6-phosphate, fructose 1,6-bisphosphate, and glyceraldehyde 3-phosphate did not differ among groups. No matched annotated features were detected for 1,3-bisphosphoglycerate, 3-phosphohydroxypyruvate, or 3-phosphoserine in the untargeted LC–MS dataset.

Across the 18 matched animals, *Pgk1* expression had positive exploratory correlations with 3PG (Pearson r = 0.369, *p* = 0.131; Spearman ρ = 0.346, *p* = 0.160) and L-serine (Pearson r = 0.357, *p* = 0.146; Spearman ρ = 0.358, *p* = 0.145), although the correlations did not reach statistical significance (Figure 2C). The remaining gene–metabolite correlations are shown in Figure 2C.

### 3.3. Hypoxia and CA Alter PGK1 Expression and Extracellular 3PG/L-Serine in HSkMC Cultures

**Figure 3:**
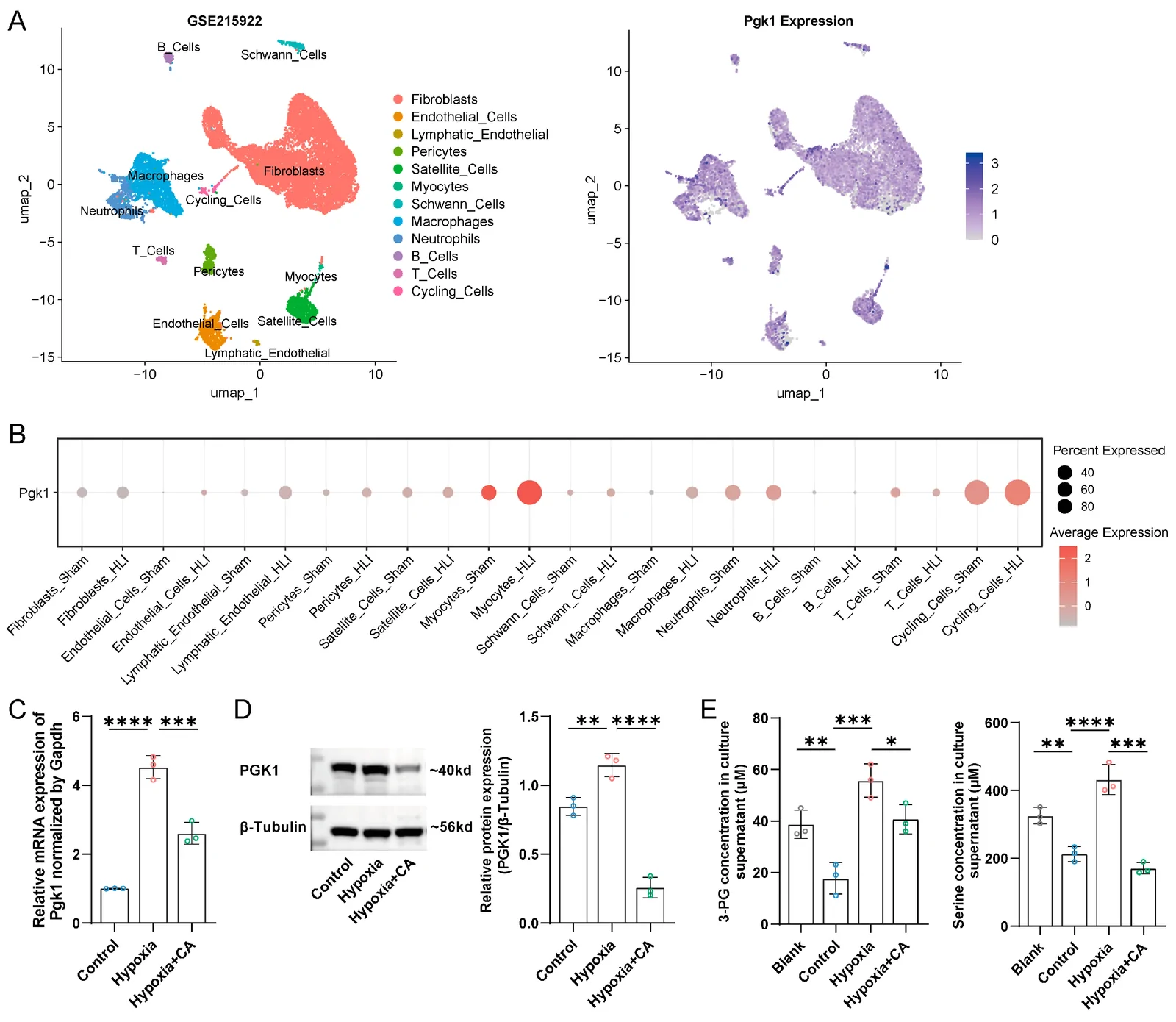
Cellular distribution of *Pgk1* and effects of hypoxia and coniferaldehyde in human skeletal muscle cells. (**A**) Uniform manifold approximation and projection (UMAP) of 15,958 cells from public gastrocnemius single-cell RNA-sequencing dataset GSE215922, colored by cell annotation (left) and normalized *Pgk1* expression (right). (**B**) Mean normalized *Pgk1* expression (color) and percentage of *Pgk1*-positive cells (point size) by cell type and condition. (**C**) Relative *PGK1* mRNA abundance normalized to *GAPDH* in normoxic control, hypoxic, and hypoxic+CA human skeletal muscle cells (HSkMCs). (**D**) Representative PGK1 and β-tubulin immunoblots and densitometry. (**E**) 3PG and serine concentrations in blank medium and HSkMC supernatants. Cell experiments were performed with three independent biological replicates, and data are presented as mean ± SD. Ordinary one-way analysis of variance followed by Dunnett’s multiple-comparisons test was used. * *p* < 0.05; ** *p* < 0.01; *** *p* < 0.001; **** *p* < 0.0001.

To examine the cellular distribution of *Pgk1*, we reanalyzed GSE215922 (Figure 3A,B). *Pgk1* was detected across multiple cell populations. In the residual Myocyte cluster, mean normalized *Pgk1* expression and the positive-cell fraction were 1.927 and 92.1% in Sham and 1.283 and 65.2% in HLI, respectively. Cycling cells showed a modest increase in mean expression from 0.993 in Sham to 1.218 in HLI and in the positive-cell fraction from 92.6% to 96.1%.

We next examined PGK1 in cultured HSkMCs. Hypoxia increased *PGK1* mRNA relative to normoxic control (*p* < 0.0001), and 5 µM CA reduced this increase (*p* < 0.001; Figure 3C). Western blotting showed the same pattern at the protein level: PGK1 was higher under hypoxia than normoxia (*p* < 0.01) and lower with CA than hypoxia alone (*p* < 0.0001; Figure 3D).

ELISA measurements showed that the 3PG concentration in culture supernatant was lower after normoxic HSkMC culture than in blank medium (*p* < 0.01), higher after hypoxia than normoxia (*p* < 0.001), and lower after hypoxia+CA than hypoxia alone (*p* < 0.05). Serine showed the same directional pattern, with lower concentrations after normoxic culture than in blank medium (*p* < 0.01), higher concentrations after hypoxia than normoxia (*p* < 0.0001), and lower concentrations after hypoxia+CA than hypoxia alone (*p* < 0.001; Figure 3E). Together, these data indicate that hypoxia increases HSkMC PGK1 and extracellular 3PG/L-serine, whereas CA reverses this response.

### 3.4. Structural Modeling and CETSA Support a Potential Interaction Between CA and PGK1

**Figure 4:**
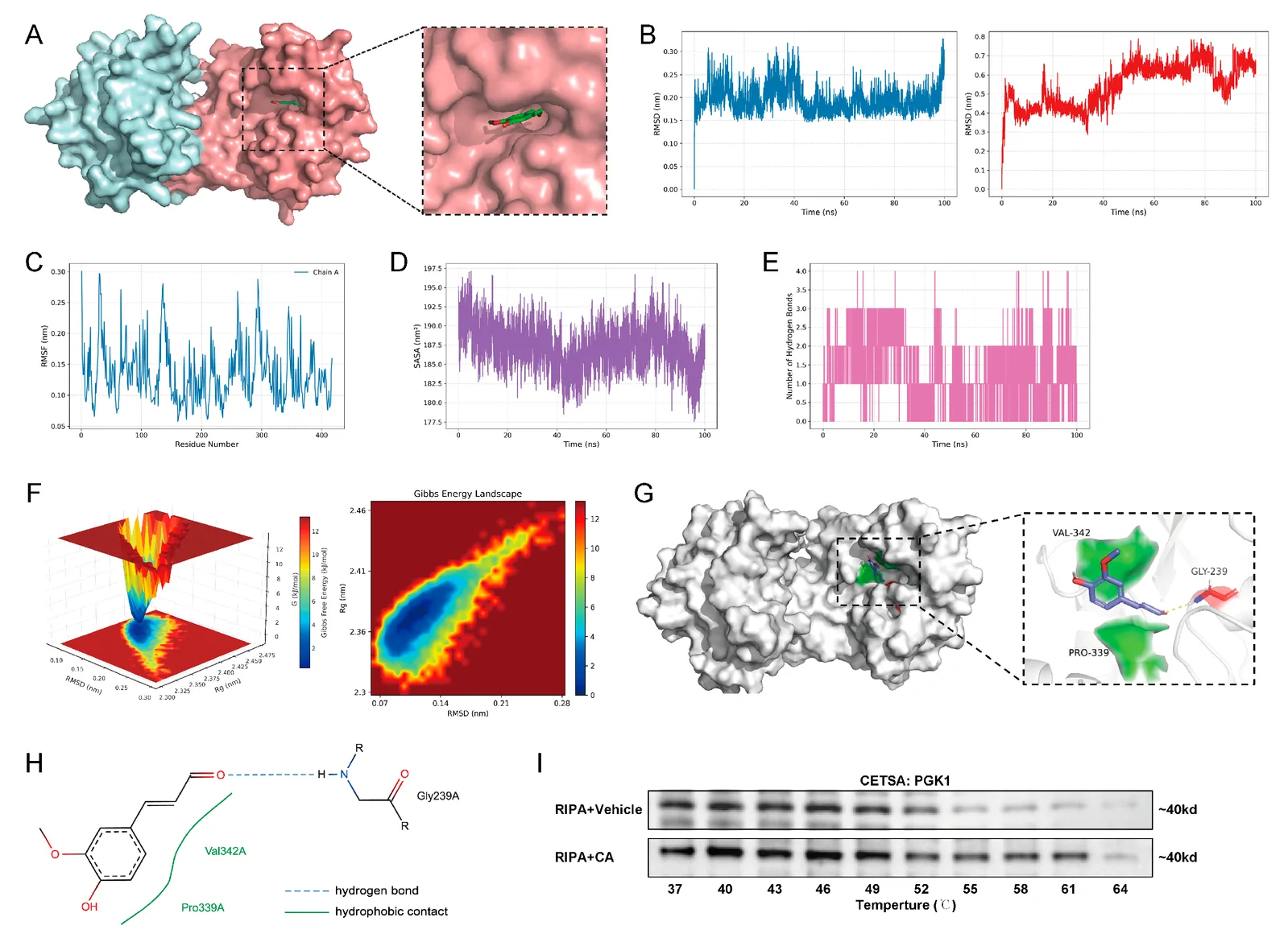
Structural modeling, molecular dynamics simulation, and cellular thermal shift assay of a potential CA–PGK1 interaction. (**A**) Highest-scoring AlphaFold3-predicted PGK1–CA complex and enlarged binding pocket. (**B**) Root-mean-square deviation (RMSD) trajectories of the PGK1 backbone (left) and ligand (right) during the 100 ns simulation. (**C**) Residue-wise root-mean-square fluctuation (RMSF). (**D**) Solvent-accessible surface area (SASA). (**E**) Number of protein–ligand hydrogen bonds. (**F**) Three-dimensional and two-dimensional free-energy landscapes calculated from RMSD and radius of gyration. (**G**) Representative lowest-energy binding conformation. (**H**) Predicted hydrogen-bond and hydrophobic contacts involving Gly239, Pro339, and Val342. (**I**) Cellular thermal shift assay (CETSA) of soluble PGK1 in vehicle- and CA-treated lysates over a 37–64 °C temperature gradient. CA, coniferaldehyde; PGK1, phosphoglycerate kinase 1.

Given the marked reduction in total PGK1 protein after CA treatment (Figure 3D), we next used AlphaFold3 to explore a potential CA–PGK1 interaction. Ten random seeds generated 50 candidate conformations, and the highest-scoring structure placed CA within a predicted PGK1 binding pocket (Figure 4A).

In a 100 ns molecular dynamics simulation, the complex fluctuated during the first approximately 40 ns and subsequently reached a relatively stable state (Figure 4B). During the 40–100 ns interval, the mean backbone RMSD was 0.188 ± 0.025 nm, and the mean ligand RMSD was 0.620 ± 0.063 nm. The mean protein RMSF was 0.134 nm, the radius of gyration was 2.383 ± 0.022 nm, the SASA was 186.6 ± 2.6 nm^2^, and approximately 1.1 protein–ligand hydrogen bonds were maintained per frame (Figure 4B–E). Free-energy-landscape analysis identified a principal low-energy conformation at an RMSD of approximately 0.135 nm and a radius of gyration of approximately 2.374 nm (Figure 4F,G). In this predicted binding pose, CA formed a hydrogen bond with Gly239 and hydrophobic contacts with Pro339 and Val342 (Figure 4H).

CETSA further showed that PGK1 signal decreased as temperature increased from 37 to 64 °C, whereas CA-treated lysates retained stronger soluble PGK1 signal than vehicle-treated lysates at approximately 55–61 °C (Figure 4I). This CA-associated thermal shift in lysate, together with the structural modeling, supports a potential interaction between CA and PGK1.

### 3.5. Conditioned Medium from CA-Treated HSkMCs Improves HUVEC Function, Whereas 3PG Supplementation Reverses These Effects

**Figure 5:**
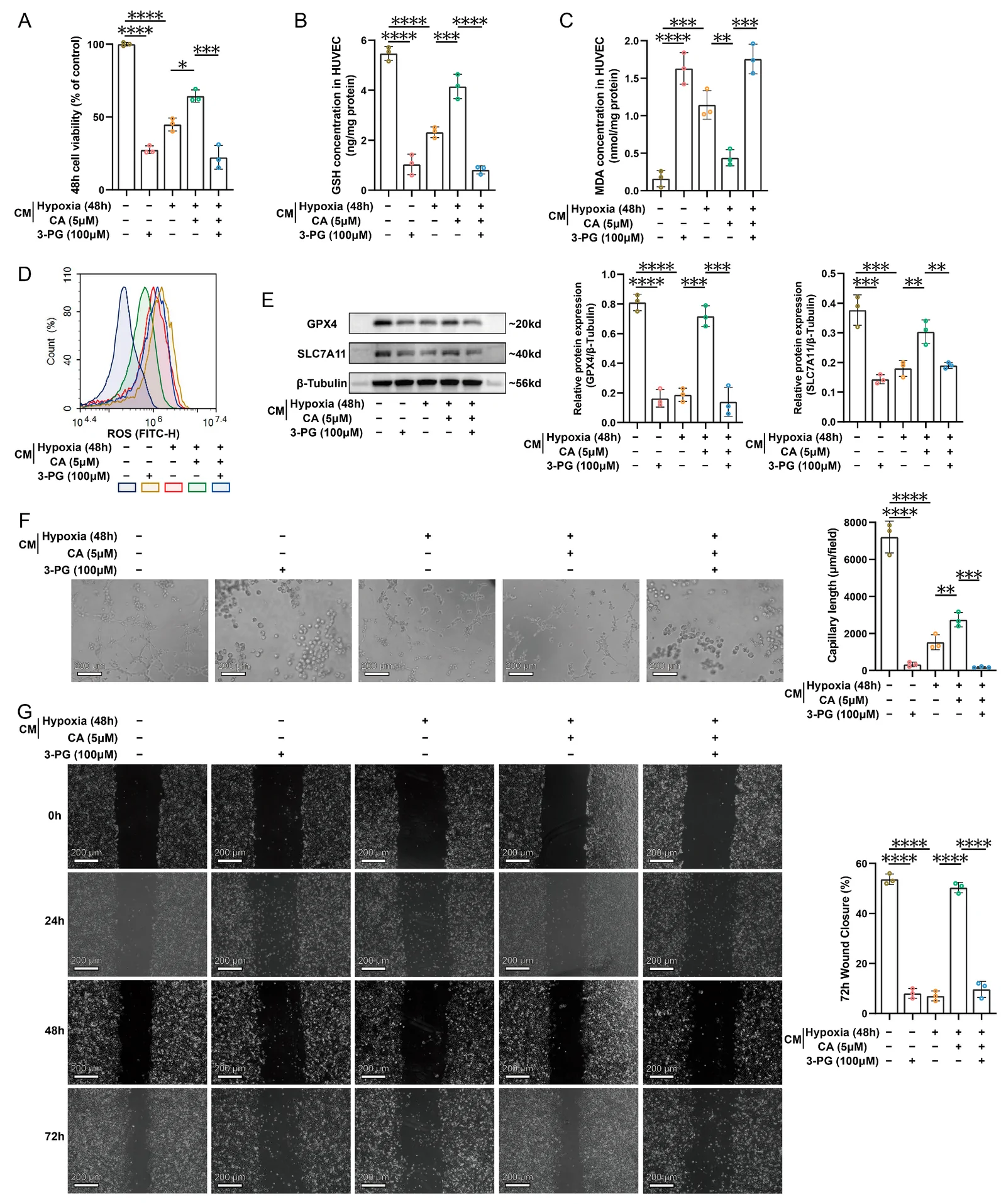
HSkMC-conditioned medium generated with coniferaldehyde improves HUVEC function, whereas 3PG supplementation reverses these effects. HUVECs were exposed to a 1:1 mixture of human skeletal muscle cell (HSkMC) conditioned medium (CM) and complete HUVEC medium. Donor HSkMCs were cultured under normoxia, hypoxia, or hypoxia with 5 µM coniferaldehyde (CA). Where indicated, 3PG was added directly to the HUVEC recipient mixture at 100 µM. (**A**) CCK-8 metabolic activity assay after 48 h. (**B**) Reduced glutathione (GSH). (**C**) Malondialdehyde (MDA). (**D**) Representative flow cytometric histograms of intracellular ROS-associated DCF fluorescence. (**E**) Representative GPX4, SLC7A11, and β-tubulin immunoblots and densitometry normalized to β-tubulin. (**F**) Representative tube-formation images and total tube length. (**G**) Representative scratch-assay images at 0, 24, 48, and 72 h and wound closure at 72 h. Scale bars in F and G, 200 µm. Experiments were performed with three independent biological replicates; quantitative data are presented as mean ± SD, whereas panel D shows representative fluorescence distributions. Tube-formation and scratch measurements were averaged over three randomly selected fields per replicate. Ordinary one-way analysis of variance followed by Dunnett’s multiple-comparisons test was used. * *p* < 0.05; ** *p* < 0.01; *** *p* < 0.001; **** *p* < 0.0001.

We next examined the effects of HSkMC-derived CM on HUVEC function. HUVECs were treated with a 1:1 mixture of HSkMC CM and complete HUVEC medium, with 100 µM 3PG added directly to the recipient mixture where indicated (Figure 5). In the CCK-8 assay, normoxic-donor CM plus 3PG and hypoxic-donor CM reduced HUVEC CCK-8 signal relative to normoxic-donor CM (both *p* < 0.0001). CM from CA-treated hypoxic HSkMCs increased the CCK-8 signal relative to hypoxic-donor CM (*p* < 0.05), whereas 3PG supplementation reduced the CCK-8 signal relative to the CA-treated condition (*p* < 0.001; Figure 5A).

GSH decreased after treatment with normoxic-donor CM plus 3PG or hypoxic-donor CM (both *p* < 0.0001). CM from CA-treated hypoxic HSkMCs increased GSH relative to hypoxic-donor CM (*p* < 0.001), and 3PG supplementation reversed this effect (*p* < 0.0001; Figure 5B). MDA showed the opposite pattern, increasing with 3PG-supplemented normoxic-donor CM and hypoxic-donor CM, decreasing with CA-treated hypoxic-donor CM, and increasing again after 3PG supplementation (Figure 5C). These findings indicate that CM from CA-treated hypoxic HSkMCs improved HUVEC redox balance, whereas 3PG supplementation reversed this effect. Representative ROS flow cytometry profiles showed increased DCF fluorescence with normoxic-donor CM plus 3PG and hypoxic-donor CM, a lower signal with CA-treated hypoxic-donor CM, and an increase after 3PG supplementation (Figure 5D). GPX4 and SLC7A11 protein levels followed the opposite pattern: both decreased with normoxic-donor CM plus 3PG or hypoxic-donor CM, increased with CA-treated hypoxic-donor CM, and decreased again after 3PG supplementation (Figure 5E).

Hypoxic-donor CM reduced HUVEC tube formation, whereas CM from CA-treated hypoxic HSkMCs increased total tube length; 3PG supplementation reversed the CA-associated improvement (Figure 5F). Similarly, 72 h wound closure was lower with hypoxic-donor CM, improved with CM from CA-treated hypoxic HSkMCs, and decreased again after 3PG supplementation (Figure 5G). Together, these results indicate that CM from CA-treated HSkMCs is associated with improved HUVEC readouts and that direct 3PG supplementation reverses these effects.

## 4. Discussion

The present study describes CA-associated changes in PGK1 and selected metabolites in ischemic skeletal muscle and links the HSkMC-derived environment to endothelial responses. In PAD, functional impairment reflects not only arterial obstruction but also mitochondrial, oxidative, and structural abnormalities in skeletal muscle [4,5]. Experimental enhancement of PFKFB3-dependent glycolysis improves muscle and perfusion outcomes after HLI [6], while hypoxia-inducible factors promote glycolytic adaptation and can induce PGK1 [21,22,23,24]. The coordinated HLI- and CA-associated changes in *Pgk1*, 3PG, and L-serine, together with the HSkMC supernatant results, are therefore consistent with a candidate hypoxia-sensitive glycolytic branch.

The recipient-cell experiment suggests that 3PG can influence endothelial phenotypes in this model. Adding 3PG to normoxic-donor CM lowered the HUVEC CCK-8 signal and GSH, increased MDA and ROS, reduced GPX4 and SLC7A11, and impaired tube formation and wound closure; adding 3PG to CM from CA-treated hypoxic HSkMCs reversed the CA-associated improvements. This pattern is consistent with endothelial oxidative injury; ferroptosis is one possible interpretation, but these redox and protein changes do not establish it. Cross-tissue studies provide relevant mechanistic precedent: in male-mouse acute kidney injury, PGK1 promoted pyruvate kinase M2/arachidonate 12-lipoxygenase-dependent ferroptosis in renal tubular epithelial cells, and systemic 3PG aggravated renal injury and lipid peroxidation [42]; in cardiac hypertrophy, cardiomyocyte PGK1 promoted vimentin/phosphoinositide 3-kinase/protein kinase B signaling and ferroptosis, while 3PG infusion worsened hypertrophy [43]. These studies support a PGK1/3PG–ferroptosis relationship outside the vascular endothelium, whereas confirmation in HUVECs still requires lipid-ROS-specific and labile-iron measurements and ferroptosis-inhibitor or iron-chelator rescue experiments.

Serine may influence vascular repair through a distinct and context-dependent route. In other cellular contexts, serine can suppress ferroptosis: mitochondrial serine hydroxymethyltransferase 2 (SHMT2)–methylenetetrahydrofolate dehydrogenase 2 (MTHFD2)-dependent serine catabolism supports NAD(P)H production, redox balance, and resistance to ferroptosis in hematopoietic stem cells, and exogenous serine protected against irradiation-induced myelosuppressive injury [44]; serine supplementation also suppressed granulosa-cell ferroptosis and preserved ovarian function in cyclophosphamide-treated mice [45]. These findings suggest that serine may influence endothelial injury through mechanisms distinct from the direct 3PG-sensitive route. In a distinct immune context, activated macrophages use serine metabolism to support interleukin-1β (IL-1β) production through serine-derived glycine and glutathione [46], while de novo serine synthesis can sustain IL-1β transcription and maturation through NAD+-dependent protein acetylation and NLR family pyrin domain containing 3 (NLRP3) inflammasome regulation [47]. Together with evidence that myeloid metabolic reprogramming and M1-like inflammatory polarization influence endothelial revascularization after HLI [20], we hypothesize that serine may influence endothelial injury indirectly through an IL-1β-rich inflammatory macrophage program in ischemic muscle; this route was not tested here and does not exclude direct endothelial effects.

The CA–PGK1 results provide a possible molecular framework linking these two metabolite routes. The CETSA shift in lysates is compatible with a possible CA–PGK1 interaction, while structural modeling offers a testable binding pose. Because total PGK1 protein decreased after CA treatment in intact HSkMCs (Figure 3D), the CETSA result reflects CA-associated thermal stabilization in lysate rather than increased cellular PGK1 abundance; whether CA alters PGK1 degradation remains to be determined. CA has also been linked to Nrf2/heme oxygenase-1 (HO-1)-dependent antioxidant and anti-inflammatory signaling in nonvascular models [32,33,34,35], which could complement PGK1-linked metabolic regulation. Together, the findings motivate a working model in which 3PG-sensitive endothelial injury and a hypothesized serine-related immune route may contribute differently to vascular repair.

This study has several limitations. The public single-cell dataset contained one pooled library per condition and relatively few cells in the Myocyte cluster, which limits interpretation of the myocyte-level *Pgk1* estimates and precludes animal-level treatment inference from individual cells. DCFH-DA fluorescence is a nonspecific oxidative readout. The ROS and GPX4/SLC7A11 findings complement the GSH/MDA results but, without lipid-ROS-specific or labile-iron measurements and pharmacological rescue, do not establish ferroptosis. Serine was not directly examined in HUVECs in the recipient-cell experiment, and macrophage and IL-1β measurements were not included; accordingly, the proposed immune route remains a literature-supported working hypothesis. Targeted metabolomics, isotope tracing, PGK1 perturbation, ferroptosis-specific assays with rescue experiments, HSkMC–macrophage–endothelial co-culture studies, and orthogonal binding assays would help clarify causality and assess whether 3PG and serine have distinct effects in the ischemic vascular microenvironment. Small sample sizes and observational omics associations limit precision and causal inference; PGK1-dependent metabolic flux and in vivo mediation remain unverified.

Extracellular 3PG and serine concentrations reflect the net balance of medium utilization, uptake, release, and donor-cell number or viability, rather than intracellular flux alone. Neither 1,3-bisphosphoglycerate nor metabolic flux was measured; lower PGK1 expression therefore cannot be assumed to account for lower 3PG. The MSI level 2 annotations and commercial-kit measurements do not replace targeted LC–MS/MS confirmation with authentic standards. PBS washing and a subsequent collection period do not by themselves exclude residual CA effects on HUVECs, and the CM experiments do not isolate metabolite-mediated effects from other medium components. The absence of a hypoxic-CM-plus-3PG condition and a dose–response series limits interpretation of the single 100 µM supplementation condition and its physiological relevance. CCK-8 measures metabolic activity rather than cell number directly, and proliferation may contribute to scratch closure over 72 h. Finally, CA administration began before HLI induction, so these findings do not establish efficacy against established ischemia or prove that the candidate metabolic axis mediates the perfusion response.

## 5. Conclusions

In this preconditioning model, CA was associated with lower *Pgk1* expression and nominal reductions in 3PG and L-serine in ischemic muscle, while CA also lowered extracellular 3PG and serine in hypoxic HSkMC cultures. Structural modeling and CETSA supported a potential CA–PGK1 interaction. In the HSkMC-conditioned-medium model, 3PG supplementation impaired HUVEC redox and angiogenic phenotypes and reversed the CA-associated improvements, supporting 3PG-sensitive endothelial responses in this model. These findings motivate further study of muscle–endothelial metabolic interactions, but PGK1 dependence, mediation of the in vivo vascular effect, and efficacy after established ischemia remain unproven.

## Figures and Tables

**Table 1:** Core KEGG pathway alterations in HLI versus Sham gastrocnemius muscle metabolomics.

KEGG Pathway	Hits (H/L)	Rich Factor	DA Score	*p* Value	BH-FDR
Glycine, serine and threonine metabolism	5 (3/2)	10.4%	0.20	9.00 × 10^−4^	0.0432
Biosynthesis of amino acids	8 (3/5)	6.3%	−0.25	9.00 × 10^−4^	0.0432
Arginine and proline metabolism	5 (3/2)	7.0%	0.20	0.0054	0.1646
Central carbon metabolism in cancer	3 (1/2)	10.0%	−0.33	0.0119	0.2735
ABC transporters	5 (4/1)	5.4%	0.60	0.0163	0.3002
Cysteine and methionine metabolism	4 (3/1)	5.9%	0.50	0.0232	0.3423
Carbon metabolism	5 (2/3)	4.5%	−0.20	0.0323	0.3423
Sulfur metabolism	3 (2/1)	6.7%	0.33	0.0350	0.3423
Biosynthesis of cofactors	10 (4/6)	3.0%	−0.20	0.0364	0.3423
Tryptophan metabolism	4 (3/1)	4.8%	0.50	0.0454	0.3423

Notes: Pathways are ranked by BH-FDR and then by raw *p* value. Rich factor is the number of differential-metabolite hits divided by the total number of annotated compounds in the pathway. Hits (H/L) reports total hits, followed by the numbers higher and lower in HLI, respectively. DA score = (higher hits − lower hits)/total hits; positive values indicate a predominance of metabolites higher in HLI, whereas negative values indicate a predominance lower in HLI. *p* values are raw pathway-enrichment *p* values. BH-FDR values are Benjamini–Hochberg-adjusted *p* values. Abbreviations: BH-FDR, Benjamini–Hochberg false-discovery rate; DA, differential abundance; HLI, hindlimb ischemia; KEGG, Kyoto Encyclopedia of Genes and Genomes.

## Data Availability

The transcriptomic and metabolomic expression matrices underlying the reported analyses are provided in Supplementary Data S1–S3. The public single-cell RNA-sequencing dataset is available from the Gene Expression Omnibus under accession GSE215922. Analysis scripts and derived summary tables are available from the corresponding authors upon reasonable request.

## Supplementary Materials

The following supporting information can be downloaded at: https://www.mdpi.com/article/10.3390/metabo16100756/s1, Figure S1: Coniferaldehyde improves hindlimb perfusion and ischemia scores in HLI mice; Figure S2: Principal component analysis (PCA) of gastrocnemius metabolite abundance in Sham, hindlimb ischemia (HLI), and HLI treated with coniferaldehyde (HLI+CA) mice; Figure S3: Principal component analysis (PCA) of gastrocnemius transcriptomic profiles in Sham, hindlimb ischemia (HLI), and HLI treated with coniferaldehyde (HLI+CA) mice; Supplementary Data S1: Transcriptomic log2(TPM + 1) expression matrix; Supplementary Data S2: Untargeted-metabolomics raw-intensity and log2(intensity + 1) matrix; Supplementary Data S3: Matched mouse, transcriptomic, and metabolomic sample metadata.

## Abbreviations

3PG, 3-phosphoglycerate; BH-FDR, Benjamini–Hochberg false-discovery rate; CA, coniferaldehyde; CI, confidence interval; CETSA, cellular thermal shift assay; CM, conditioned medium; GAP, glyceraldehyde 3-phosphate; GPX4, glutathione peroxidase 4; GSH, reduced glutathione; HLI, hindlimb ischemia; IL-1β, interleukin-1β; HSkMC, human skeletal muscle cell; HUVEC, human umbilical vein endothelial cell; KEGG, Kyoto Encyclopedia of Genes and Genomes; MD, molecular dynamics; MDA, malondialdehyde; Nrf2, nuclear factor erythroid 2-related factor 2; PAD, peripheral artery disease; PCA, principal component analysis; PERMDISP, permutational analysis of multivariate dispersion; PERMANOVA, permutational multivariate analysis of variance; PGK1, phosphoglycerate kinase 1; PHGDH, phosphoglycerate dehydrogenase; PLS-DA, partial least-squares discriminant analysis; PSAT1, phosphoserine aminotransferase 1; PSPH, phosphoserine phosphatase; qRT-PCR, quantitative reverse-transcription polymerase chain reaction; RMSD, root-mean-square deviation; RMSF, root-mean-square fluctuation; SASA, solvent-accessible surface area; ROS, reactive oxygen species; SD, standard deviation; SLC7A11, solute carrier family 7 member 11; UMAP, uniform manifold approximation and projection.

## References

[1] Gornik H.L., Aronow H.D., Goodney P.P., Arya S., Brewster L.P., Byrd L., Chandra V., Drachman D.E., Eaves J.M., Ehrman J.K. (2024). 2024 ACC/AHA/AACVPR/APMA/ABC/SCAI/SVM/SVN/SVS/SIR/VESS guideline for the management of lower extremity peripheral artery disease: A report of the American College of Cardiology/American Heart Association Joint Committee on Clinical Practice Guidelines. Circulation.

[2] Kim M.S., Hwang J., Yon D.K., Lee S.W., Jung S.Y., Park S., Johnson C.O., Stark B., Razo C., Abbasian M. (2023). Global burden of peripheral artery disease and its risk factors, 1990–2019: A systematic analysis for the Global Burden of Disease Study 2019. Lancet Glob. Health.

[3] Aday A.W., Matsushita K. (2021). Epidemiology of Peripheral Artery Disease and Polyvascular Disease. Circ. Res..

[4] Hiatt W.R., Armstrong E.J., Larson C.J., Brass E.P. (2015). Pathogenesis of the limb manifestations and exercise limitations in peripheral artery disease. Circ. Res..

[5] Ferrucci L., Candia J., Ubaida-Mohien C., Lyashkov A.E., Banskota N., Leeuwenburgh C., Wohlgemuth S.E., Guralnik J.M., Kaileh M., Zhang D. (2023). Transcriptomic and proteomic of gastrocnemius muscle in peripheral artery disease. Circ. Res..

[6] Ryan T.E., Schmidt C.A., Tarpey M.D., Amorese A.J., Yamaguchi D.J., Goldberg E.J., Iñigo M.R., Karnekar R., O’Rourke A.R., Ervasti J.M. (2020). PFKFB3-mediated glycolysis rescues myopathic outcomes in the ischemic limb. JCI Insight.

[7] Inampudi C., Akintoye E., Ando T., Briasoulis A. (2018). Angiogenesis in peripheral arterial disease. Curr. Opin. Pharmacol..

[8] Song Y., Yang J., Li T., Sun X., Lin R., He Y., Sun K., Han J., Yang G., Li X. (2023). CD34+ cell-derived fibroblast–macrophage cross-talk drives limb ischemia recovery through the OSM–ANGPTL signaling axis. Sci. Adv..

[9] Carmeliet P., Jain R.K. (2011). Molecular mechanisms and clinical applications of angiogenesis. Nature.

[10] Potente M., Gerhardt H., Carmeliet P. (2011). Basic and therapeutic aspects of angiogenesis. Cell.

[11] Eelen G., de Zeeuw P., Treps L., Harjes U., Wong B.W., Carmeliet P. (2018). Endothelial cell metabolism. Physiol. Rev..

[12] Li X., Sun X., Carmeliet P. (2019). Hallmarks of endothelial cell metabolism in health and disease. Cell Metab..

[13] Pasut A., Lama E., Van Craenenbroeck A.H., Kroon J., Carmeliet P. (2025). Endothelial cell metabolism in cardiovascular physiology and disease. Nat. Rev. Cardiol..

[14] De Bock K., Georgiadou M., Schoors S., Kuchnio A., Wong B.W., Cantelmo A.R., Quaegebeur A., Ghesquière B., Cauwenberghs S., Eelen G. (2013). Role of PFKFB3-driven glycolysis in vessel sprouting. Cell.

[15] Schoors S., De Bock K., Cantelmo A.R., Georgiadou M., Ghesquière B., Cauwenberghs S., Kuchnio A., Wong B.W., Quaegebeur A., Goveia J. (2014). Partial and transient reduction of glycolysis by PFKFB3 blockade reduces pathological angiogenesis. Cell Metab..

[16] Rohlenova K., Goveia J., García-Caballero M., Subramanian A., Kalucka J., Treps L., Falkenberg K.D., de Rooij L.P.M.H., Zheng Y., Lin L. (2020). Single-cell RNA sequencing maps endothelial metabolic plasticity in pathological angiogenesis. Cell Metab..

[17] Vandekeere S., Dubois C., Kalucka J., Sullivan M.R., García-Caballero M., Goveia J., Chen R., Diehl F.F., Bar-Lev L., Souffreau J. (2018). Serine synthesis via PHGDH is essential for heme production in endothelial cells. Cell Metab..

[18] Arany Z., Foo S.Y., Ma Y., Ruas J.L., Bommi-Reddy A., Girnun G., Cooper M., Laznik D., Chinsomboon J., Rangwala S.M. (2008). HIF-independent regulation of VEGF and angiogenesis by the transcriptional coactivator PGC-1α. Nature.

[19] Turiel G., Desgeorges T., Masschelein E., Fan Z., Lussi D., Capelle C.M., Bernardini G., Ardicoglu R., Schönberger K., Birrer M. (2025). Single-cell compendium of muscle microenvironment in peripheral artery disease reveals altered endothelial diversity and LYVE1+ macrophage activation. Nat. Cardiovasc. Res..

[20] Yadav S., Ganta V.C., Varadarajan S., Ong V., Shi Y., Das A., Ash D., Nagarkoti S., McMenamin M., Kelley S. (2025). Myeloid DRP1 deficiency limits revascularization in ischemic muscles via inflammatory macrophage polarization and metabolic reprogramming. JCI Insight.

[21] Semenza G.L. (2012). Hypoxia-inducible factors in physiology and medicine. Cell.

[22] Kim J.W., Tchernyshyov I., Semenza G.L., Dang C.V. (2006). HIF-1-mediated expression of pyruvate dehydrogenase kinase: A metabolic switch required for cellular adaptation to hypoxia. Cell Metab..

[23] Kierans S.J., Taylor C.T. (2021). Regulation of glycolysis by the hypoxia-inducible factor (HIF): Implications for cellular physiology. J. Physiol..

[24] Li H., Ko H.P., Whitlock J.P. (1996). Induction of phosphoglycerate kinase 1 gene expression by hypoxia. J. Biol. Chem..

[25] Locasale J.W., Grassian A.R., Melman T., Lyssiotis C.A., Mattaini K.R., Bass A.J., Heffron G.J., Metallo C.M., Muranen T., Sharfi H. (2011). Phosphoglycerate dehydrogenase diverts glycolytic flux and contributes to oncogenesis. Nat. Genet..

[26] Possemato R., Marks K.M., Shaul Y.D., Pacold M.E., Kim D., Birsoy K., Sethumadhavan S., Woo H.K., Jang H.G., Jha A. (2011). Functional genomics reveal that the serine synthesis pathway is essential in breast cancer. Nature.

[27] Yang M., Vousden K.H. (2016). Serine and one-carbon metabolism in cancer. Nat. Rev. Cancer.

[28] Mattaini K.R., Sullivan M.R., Vander Heiden M.G. (2016). The importance of serine metabolism in cancer. J. Cell Biol..

[29] Ducker G.S., Rabinowitz J.D. (2017). One-Carbon Metabolism in Health and Disease. Cell Metab..

[30] Ye J., Fan J., Venneti S., Wan Y.W., Pawel B.R., Zhang J., Finley L.W.S., Lu C., Lindsten T., Cross J.R. (2014). Serine catabolism regulates mitochondrial redox control during hypoxia. Cancer Discov..

[31] Li X., Jiang Y., Meisenhelder J., Yang W., Hawke D.H., Zheng Y., Xia Y., Aldape K., He J., Hunter T. (2016). Mitochondria-translocated PGK1 functions as a protein kinase to coordinate glycolysis and the TCA cycle in tumorigenesis. Mol. Cell.

[32] Dong Y., Stewart T., Bai L., Li X., Xu T., Iliff J.J., Shi M., Zheng D., Yuan L., Wei T. (2020). Coniferaldehyde attenuates Alzheimer’s pathology via activation of Nrf2 and its targets. Theranostics.

[33] Chen H.H., Wang T.C., Lee Y.C., Shen P.T., Chang J.Y., Yeh T.K., Huang C.H., Chang H.H., Cheng S.Y., Lin C.Y. (2015). Novel Nrf2/ARE activator, trans-coniferylaldehyde, induces a HO-1-mediated defense mechanism through a dual p38α/MAPKAPK-2 and PK-N3 signaling pathway. Chem. Res. Toxicol..

[34] Kim K.M., Heo D.R., Kim Y.A., Lee J., Kim N.S., Bang O.S. (2016). Coniferaldehyde inhibits LPS-induced apoptosis through the PKC α/βII/Nrf2/HO-1-dependent pathway in RAW264.7 macrophage cells. Environ. Toxicol. Pharmacol..

[35] Park J.M., Park J.E., Park J.S., Leem Y.H., Kim D.Y., Hyun J.W., Kim H.S. (2024). Anti-inflammatory and antioxidant mechanisms of coniferaldehyde in lipopolysaccharide-induced neuroinflammation: Involvement of AMPK/Nrf2 and TAK1/MAPK/NF-κB signaling pathways. Eur. J. Pharmacol..

[36] Sumner L.W., Amberg A., Barrett D., Beale M.H., Beger R., Daykin C.A., Fan T.W.-M., Fiehn O., Goodacre R., Griffin J.L. (2007). Proposed minimum reporting standards for chemical analysis: Chemical Analysis Working Group (CAWG), Metabolomics Standards Initiative (MSI). Metabolomics.

[37] Ritchie M.E., Phipson B., Wu D., Hu Y., Law C.W., Shi W., Smyth G.K. (2015). Limma powers differential expression analyses for RNA-sequencing and microarray studies. Nucleic Acids Res..

[38] Rohart F., Gautier B., Singh A., Lê Cao K.-A. (2017). mixOmics: An R package for ‘omics feature selection and multiple data integration. PLoS Comput. Biol..

[39] Anderson M.J. (2001). A new method for non-parametric multivariate analysis of variance. Austral Ecol..

[40] Benjamini Y., Hochberg Y. (1995). Controlling the false discovery rate: A practical and powerful approach to multiple testing. J. R. Stat. Soc. Ser. B Methodol..

[41] Hao Y., Hao S., Andersen-Nissen E., Mauck W.M., Zheng S., Butler A., Lee M.J., Wilk A.J., Darby C., Zager M. (2021). Integrated analysis of multimodal single-cell data. Cell.

[42] Zhu X.-X., Meng X.-Y., Zhang A.-Y., Zhao C.-Y., Wei Y.-T., Wen Y.-Y., Su J.-B., Fu X., Chen G., Xu A.-J. (2025). Inhibition of PGK1 ameliorates acute kidney injury through inactivating the PKM2/ALOX12/ferroptosis pathway in a study with male mice. Nat. Commun..

[43] Zhu X.-X., Zhang A.-Y., Xu G.-W., Li R., Gao S.-Q., Ji L.-M., Li Y.-F., He T., Wei M., Yang L. (2026). PGK1 drives cardiac hypertrophy by regulating the vimentin/PI3K/Akt pathway. Circ. Res..

[44] Du C., Liu C., Yu K., Zhang S., Fu Z., Chen X., Liao W., Chen J., Zhang Y., Wang X. (2024). Mitochondrial serine catabolism safeguards maintenance of the hematopoietic stem cell pool in homeostasis and injury. Cell Stem Cell.

[45] Gu H.-C., Zhuo Y.-Q., Wang L.-F., Zhang Y.-W., Li D.-S., Chen L.-Q., Li Z.-H., Wang Y.-Y., Wang Y.-K., Liao H.-T. (2026). Serine inhibits granulosa cell ferroptosis to maintain ovarian function. Nat. Commun..

[46] Rodriguez A.E., Ducker G.S., Billingham L.K., Martinez C.A., Mainolfi N., Suri V., Friedman A., Manfredi M.G., Weinberg S.E., Rabinowitz J.D. (2019). Serine metabolism supports macrophage IL-1β production. Cell Metab..

[47] Wang C., Chen Q., Chen S., Fan L., Gan Z., Zhao M., Shi L., Bin P., Yang G., Zhou X. (2024). Serine synthesis sustains macrophage IL-1β production via NAD+-dependent protein acetylation. Mol. Cell.

