# Supplementary material for: Coniferaldehyde Reverses PGK1–3-Phosphoglycerate–L-Serine Metabolic Reprogramming in Hindlimb Ischemia
Source: Metabolites. 2026 Oct 9;16(10):756. doi: 10.3390/metabo16100756 (PMC13707088; doi:10.3390/metabo16100756)
Supplement: Supplementary file 1 [file metabolites-16-00756-s001.zip › Supplementary_Information.pdf]

Supplementary Information

Supplementary Information for "Coniferaldehyde Reverses PGK1–3-Phosphoglycerate–L-Serine Metabolic Reprogramming in Hindlimb Ischemia"

Yun Dong, Chunbo Ma, Xueli Wang, Jiaping Han, Zina Zhu, Suwen Hu, Zixuan Hu *and Liangrong Wang*

* Correspondence: arerong1984@126.com (L.W.); zixuanhu_eric@outlook.com (Z.H.)

Contents

Figure S1. Body weight, hindlimb perfusion, and ischemia scores in the mouse HLI cohort.

Figure S2. Principal component analysis of gastrocnemius metabolite abundance.

Figure S3. Principal component analysis of gastrocnemius transcriptomic profiles.

Supplementary Data S1. Transcriptomic log2(TPM + 1) expression matrix.

Supplementary Data S2. Untargeted-metabolomics raw-intensity and log2(intensity + 1) matrix.

Supplementary Data S3. Matched mouse, transcriptomic, and metabolomic sample metadata.

Figure S1

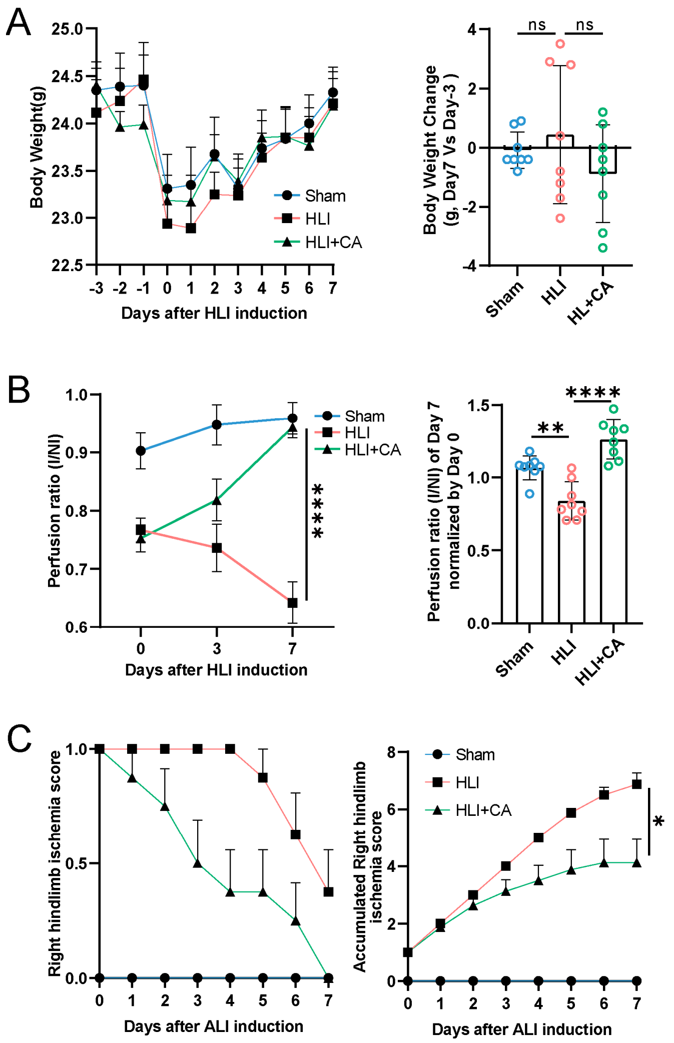

Figure S1. Coniferaldehyde improves hindlimb perfusion and ischemia scores in HLI mice. Male C57BL/6J mice underwent Sham surgery or hindlimb ischemia (HLI) and received vehicle or coniferaldehyde (CA; 20 mg/kg, intraperitoneally, once daily from Day −3 through Day 7); n = 8 mice/group. (A) Body weight from Day −3 through Day 7 and the Day-7 change relative to Day −3. (B) Ischemic/nonischemic (I/NI) laser-Doppler perfusion ratios on Days 0, 3, and 7 and the Day-7 ratio normalized to Day 0. (C) Daily right-hindlimb ischemia scores and cumulative scores through Day 7. Data are presented as mean ± SD; points in summary plots represent individual mice. **P < 0.01 and ****P < 0.0001 for the indicated perfusion comparisons; *P < 0.05 for the indicated cumulative-score comparison; ns, not significant.

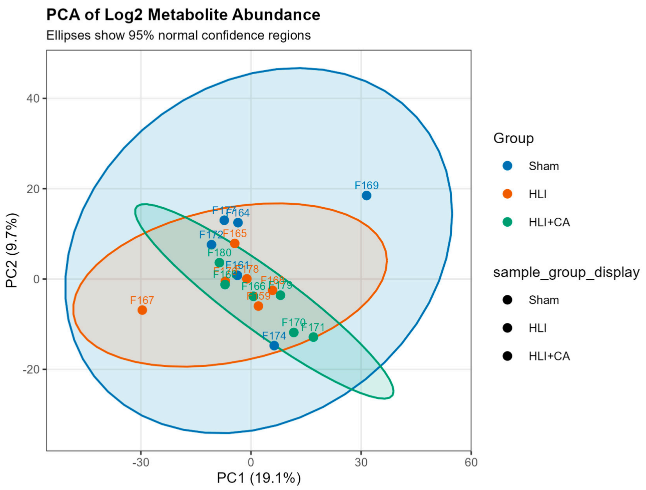

Figure S2. Principal component analysis (PCA) of gastrocnemius metabolite abundance in Sham, hindlimb ischemia (HLI), and HLI treated with coniferaldehyde (HLI+CA) mice (n = 6/group). PCA was performed on feature-wise scaled log2(intensity + 1) values for 871 annotated metabolite features. Each point represents one mouse; labels identify samples, and blue, orange, and green indicate Sham, HLI, and HLI+CA, respectively. PC1 and PC2 explain 19.1% and 9.7% of variance. Ellipses are 95% normal confidence regions and are descriptive, not tests of group separation.

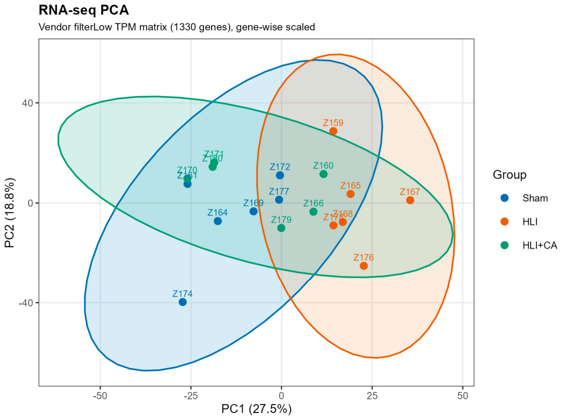

Figure S3. Principal component analysis (PCA) of gastrocnemius transcriptomic profiles in Sham, hindlimb ischemia (HLI), and HLI treated with coniferaldehyde (HLI+CA) mice (n = 6/group). PCA used gene-wise scaled transcripts-per-million (TPM) values for the 1,330 genes retained in the vendor low-expression-filtered matrix. Each point represents one mouse; labels identify RNA samples, and blue, orange, and green indicate Sham, HLI, and HLI+CA, respectively. PC1 and PC2 explain 27.5% and 18.8% of variance. Ellipses show within-group 95% normal confidence regions; the plot is an unsupervised descriptive analysis.

Supplementary Data Files

Supplementary Data S1. Transcriptomic log2(TPM + 1) expression matrix.

Supplementary Data S2. Untargeted-metabolomics raw-intensity and log2(intensity + 1) matrix.

Supplementary Data S3. Matched mouse, transcriptomic, and metabolomic sample metadata.
